# Axillary Overtreatment in Patients with Breast Cancer After Neoadjuvant Chemotherapy in the Current Era of Targeted Axillary Dissection

**DOI:** 10.3390/cancers17020178

**Published:** 2025-01-08

**Authors:** Ondřej Zapletal, Jan Žatecký, Lucie Gabrielová, Iveta Selingerová, Miloš Holánek, Petr Burkoň, Oldřich Coufal

**Affiliations:** 1 Department of Surgical Oncology, Masaryk Memorial Cancer Institute, 656 53 Brno, Czech Republic; ondrej.zapletal@mou.cz (O.Z.); gabrielova@mou.cz (L.G.); coufal@mou.cz (O.C.); 2 Department of Surgical Oncology, Faculty of Medicine, Masaryk University, 625 00 Brno, Czech Republic; 3 Department of Surgery, Silesian Hospital in Opava, 746 01 Opava, Czech Republic; 4 The Institute of Paramedical Health Studies, Faculty of Public Policies, Silesian University, 746 01 Opava, Czech Republic; 5 Research Centre for Applied Molecular Oncology (RECAMO), Masaryk Memorial Cancer Institute, 656 53 Brno, Czech Republic; iveta.selingerova@mou.cz; 6 Department of Pharmacology, Faculty of Medicine, Masaryk University, 625 00 Brno, Czech Republic; 7 Department of Comprehensive Cancer Care, Masaryk Memorial Cancer Institute, 656 53 Brno, Czech Republic; holanek@mou.cz; 8 Department of Comprehensive Cancer Care, Faculty of Medicine, Masaryk University, 625 00 Brno, Czech Republic; 9 Department of Radiation Oncology, Masaryk Memorial Cancer Institute, 656 53 Brno, Czech Republic; burkon@mou.cz; 10 Department of Radiation Oncology, Faculty of Medicine, Masaryk University, 625 00 Brno, Czech Republic

**Keywords:** axillary dissection, targeted axillary dissection, breast cancer, neoadjuvant chemotherapy

## Abstract

**Highlights:**

The study aimed to determine the proportion of breast cancer patients indicated for ALND after NAC who later show ypN0, and to identify the reasons that led to ALND which may represent surgical overtreatment. The most common reasons for potentially unnecessary ALND included: inflammatory carcinoma (*n* = 13, 29.5%), locally advanced carcinoma (*n* = 5, 11.4%), occult carcinoma (*n* = 2, 4.5%), or persistent lymphadenopathy on US examination after NAC, particularly in the tumor phenotypes HER2-positive and TNBC (*n* = 8, 18.2%).

**Simple Summary:**

This retrospective analysis defines subgroups of breast cancer patients treated with neoadjuvant chemotherapy who may suffer from surgical overtreatment in the axilla even in the current modern era of targeted axillary dissection. These include patients with inflammatory carcinoma, locally advanced carcinoma, occult carcinoma, or patients with persistent findings of suspicious pathological nodes after NAC according to ultrasound examination, especially in the tumor phenotypes HER2-positive and triple-negative breast cancer. Therefore, our study could serve as the background for multidisciplinary teams discussing the possibility of omitting axillary lymph node dissection in a well-selected subgroup of patients with breast cancer after neoadjuvant chemotherapy.

**Abstract:**

Background: In the current era of targeted axillary dissection (TAD), there are still cases where axillary lymph node dissection (ALND) is indicated, but histopathological examination confirms the regression of nodal metastases (ypN0). In this situation, ALND may represent undesirable overtreatment. Methods: A retrospective study at the Comprehensive Cancer Centre was conducted based on a prospectively maintained database. Patients who underwent surgery after neoadjuvant chemotherapy (NAC) between 2020 and 2023 were selected, specifically those for whom ALND was directly indicated after NAC. Subsequently, clinical–pathological characteristics were compared between cases with ypN0 and those with persistent metastases (ypN+). The reasons for indicating ALND in ypN0 cases were extracted from the medical records. Results: ALND was indicated in 118 cases across 117 patients, of which ypN0 was observed in 44 cases (37%). There were significantly more ypN0 cases for inflammatory carcinomas (68%), the non-luminal HER2-positive phenotype (76%), and carcinomas with histopathological regression of the primary tumor (76%) or the persistence of only the non-invasive component of ypTis (67%). Typical reasons for ALND in ypN0 cases included inflammatory carcinoma (*n* = 13, 29.5%), locally advanced carcinoma (*n* = 5, 11.4%), occult carcinoma (*n* = 2, 4.5%), or persistent lymphadenopathy on ultrasound examination after NAC, especially in the tumor phenotypes HER2-positive and triple-negative breast cancer (TNBC) (*n* = 8, 18.2%). Conclusions: Through real-world evidence data analysis, subgroups of breast cancer patients treated with NAC were identified who may experience surgical overtreatment in the axilla. These include patients with inflammatory carcinoma, locally advanced carcinoma, occult carcinoma, or patients with persistent lymphadenopathy on US examination after NAC, particularly in the tumor phenotypes HER2-positive and TNBC.

## 1. Introduction

Historically, axillary lymph node dissection (ALND) was the standard procedure for breast cancer patients after neoadjuvant chemotherapy (NAC). Subsequent research has shown that for patients initially presenting without clinical signs of axillary lymphadenopathy (cN0), it is safe to opt for sentinel lymph node biopsy (SLNB) after NAC, as confirmed by a meta-analysis [1]. The situation becomes less clear when considering patients with nodal metastases at the time of diagnosis (cN+) that clinically regress after NAC. Two completed multicentric studies have shown that SLNB alone in this situation has unacceptably high false-negative rates (FNRs) exceeding 10% [2,3]. In 2016, a paper defining a new surgical procedure called Targeted Axillary Dissection (TAD) was published [4]. A prerequisite for this procedure is marking the most prominent pathological node before neoadjuvant treatment (“clipped node”). If axillary lymphadenopathy regresses after NAC, patients undergo SLNB and the selective removal of the clipped node using iodine-125 seed localization. In the original study, this approach showed a reduction in FNR to an acceptable level of 2.0% [4]. Subsequent studies also confirm the low false negativity of this procedure [5]. Patients in whom nodes excised during TAD show histological evidence of persistent tumor are recommended to undergo the completion of ALND, whereas patients in whom the nodes are free of cancer may be spared. The main purpose of this conservative approach is to reduce morbidity while maintaining adequate oncological staging and local control. Adjuvant radiotherapy (RT) is usually indicated after TAD due to the original nodal involvement, regardless of whether nodal metastases regressed during NAC or not, since it is based on the maximal disease stage at diagnosis [6]. With the improving efficacy of systemic treatments, it can be anticipated that cases of complete histopathologic response with no need for RT will become increasingly common, provided that the initial stage of the tumor is not advanced [7]. Prospective trials on this topic are ongoing. Although the long-term oncologic outcomes of TAD are not yet reliably known and have been the subject of several ongoing studies (e.g., AXSANA) [8], this procedure is rapidly spreading into routine surgical practice in various modifications. The modifications may consist of the method of marking the nodes [9]. It is also unclear whether the procedure can be used regardless of the stage of the primary tumor or the level of initial nodal involvement. In some institutions, only the marked node is removed (Marked Lymph Node Dissection) without any additional sentinel nodes, whose added informational value appears to be low [10].

At our institution, TAD started to be cautiously used in 2017, and since the end of 2019, it has been used routinely, without clearly defined contraindications, except for inflammatory carcinomas. The pilot results were published in 2018 [11]. We follow the procedure described in the original work by Caudle et al. [4]. To label the most prominent pathological node before treatment, a clip (HYDROMARK^TM^, Devicor Medical Products, Inc., Cincinnati, OH, USA) is inserted under ultrasound guidance, usually simultaneously with a core-cut biopsy of this node to confirm metastatic involvement. After NAC, patients undergo ultrasound (US) re-staging. If there is a regression of axillary lymphadenopathy, TAD is indicated and the clipped node is marked with an iodine seed for identification during surgery. Specimen radiography is used to confirm the presence of the clip and the seed in the resection specimen. Ideally, only patients with persistent nodal involvement (ypN+) should undergo ALND after NAC in this current “modern” era. However, even nowadays, some patients undergo ALND after NAC, where histopathological examination of the specimen reveals the regression of nodal metastases (ypN0), which may represent surgical overtreatment. The aim of the study was to determine the proportion of breast cancer patients indicated for ALND after NAC who later show ypN0, to identify the reasons that led to ALND, and to identify possible predisposing clinical–pathological characteristics for this potential overtreatment.

## 2. Methods

A retrospective mono-institutional study conducted at the OECI Comprehensive Cancer Centre. From a prospectively maintained parametric database of surgical protocols, patients with breast cancer without distant metastases were initially identified who underwent neoadjuvant chemotherapy or chemotherapy combined with biological therapy and subsequently underwent surgical resection during the 4-year period from 2020 to 2023. From this group, a subgroup of cases was selected in which ALND was directly indicated immediately after NAC as the first and definitive axillary procedure. Among the selected cases, the ypN0 and ypN+ groups were identified by reviewing the results of the histopathological evaluation of the surgical specimen. Histopathological examination was conducted in accordance with the standards of the College of American Pathologists [12] applicable at the time of the surgery. Detailed clinicopathological characteristics were extracted from routine medical records and compared between the ypN0 and ypN+ groups.

The inclusion criteria were histologically verified invasive breast carcinoma or carcinoma of mammary origin in regional (axillary) lymph nodes with any cT and any cN, the absence of distant metastases at initial presentation or at any time before surgery (M0), neoadjuvant chemotherapy or chemotherapy + biological therapy, a date of surgery within the period of the calendar years 2020–2023 (4 years) and directly indicated ALND as the first procedure during therapeutic considerations before the surgery.

From the texts of diagnostic and therapeutic considerations, the reasons why ALND was indicated in ypN0 cases were determined. The identified reasons were categorized into several groups. Patient clinicopathological characteristics were described using standard summary statistics, including the median and range for continuous variables and frequencies and proportions for categorical variables. A logistic regression model was used to evaluate differences between the ypN0 and ypN+ groups and was interpreted using the odds ratios (ORs) with 95% confidence intervals (95% CI). All statistical analyses were performed using R version 4.4.0 at a significance level 0.01. The study was approved by the Institutional Clinical Research Council and the Ethics Committee (reference number 2024/412/MOU). All experiments were performed in accordance with relevant guidelines and regulations.

## 3. Results

**Figure 1:**
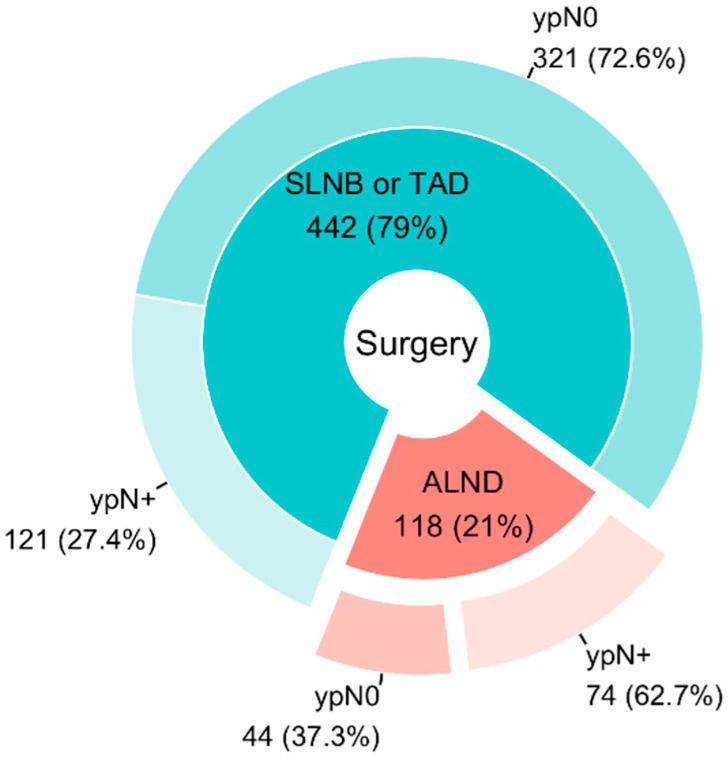
Type of surgery indicated after NAC.

During the observed period, 560 carcinomas were resected after NAC in 544 patients (15 cases involved synchronous bilateral cancer and 1 case involved metachronous bilateral cancer). ALND was directly indicated as the first and only procedure in the axilla in 118 cases in 117 patients (116 women and 1 man), with one case involving synchronous bilateral cancer. Of these 118 cases, ypN0 was observed in 44 cases (37%). In the remaining cases (*n* = 74), histopathological evidence of metastases persisted in the nodes (ypN+). The situation is illustrated in Figure 1.

The table provides detailed clinicopathological characteristics of cases with directly indicated ALND in the ypN0/ypN+ groups (Table 1). Patients with inflammatory carcinomas were more often ypN0 (68%) compared to T2 (OR = 6.5, *p* = 0.002) or T4b (OR = 7.6, *p* = 0.007). In the non-luminal HER2-positive (human epidermal growth factor receptor 2-positive) tumors, ypN0 was found in 76%, significantly more than in luminal B HER2-negative (OR = 102, *p* < 0.001) cases. Patients with a complete disappearance of the invasive component of the tumor in the breast (ypT0/ypTis) were more often ypN0 (73%) compared to patients with both ypT1 (OR = 7.6, *p* < 0.001) and ypT2–4 (OR = 46.4, *p* < 0.001) residual disease.

The reasons why ALND was indicated in ypN0 patients are listed in Table 2.

## 4. Discussion

It is well established that HER2-positive and triple-negative tumors exhibit the highest likelihood of achieving nodal histopathological regression [13]. Consistent with this, the ypN0 group in our cohort consisted almost exclusively of tumors with these two phenotypes (Table 1). Two cases of tumors with different phenotypes in the ypN0 group require further discussion. In the case of a luminal B HER2-negative tumor, it involved inflammatory carcinoma with nodal burden T4dN1. After surgery and histological examination, the classification was ypT0 ypN0. However, this did not represent a complete clinical response in the true sense (Chevalier 1) because ITC and multifocal lymphovascular invasion persisted in three nodes. In the case of Luminal A lobular carcinoma (initially classified as cTXN1), NAC was indicated due to a contralateral HER2-positive carcinoma (T3N0), and, surprisingly, the lobular carcinoma also regressed after NAC.

The assumption was also confirmed that there is a certain, albeit not absolute, correlation between achieving regression in the primary tumor and the regional lymph nodes, as previously reported [14]. In the group of breast cancers with complete pathological regression of the primary tumor after NAC, ypN0 was observed in 76% of cases. Similarly, in tumors where only the non-invasive component persisted after NAT (ypTis), ypN0 was present in 67% of cases.

Inflammatory carcinoma, i.e., T4d according to the TNM classification [15], is usually associated with nodal metastases (N+). Current standards allow only ALND, regardless of the initial nodal staging and the effect of NAC [16]. This precaution stems from the relatively rare occurrence of inflammatory carcinoma, its often-aggressive disease course, and the fact that T4d stage was typically an exclusion criterion in clinical studies aimed at reducing axillary surgical radicality. Additionally, sentinel lymph node detection has proven unsuccessful in most cases of inflammatory carcinoma [17]. However, the high number of ypN0 cases of inflammatory carcinoma in our cohort suggests that the likelihood of achieving complete histopathological regression is significant, even in this patient group, in the current era of modern systemic treatment. Among the 13 inflammatory carcinoma patients in the ypN0 group, complete radiological regression of nodal involvement was confirmed by re-staging US examination in seven cases. If not for their classification as inflammatory carcinomas, these patients would have been candidates for TAD. In the aforementioned ypN0 group, it is noteworthy that two patients had undergone RT before surgery due to incomplete clinical regression of inflammation (redness) following NAC. In both cases, the carcinoma was HER2-positive. It appears highly probable that, in terms of overall prognosis for patients with inflammatory carcinoma, the response to systemic treatment (and RT) is more crucial than surgical radicality in the axilla. Our data indicate that, in the era of TAD, there is an urgent need to reevaluate the routine indication for ALND in this patient group, especially for those with HER2-positive or triple-negative breast cancer (TNBC) phenotypes. Similar conclusions are drawn in the study by Karanlik et al. [18]. The situation is further complicated by the fact that the diagnosis of inflammatory carcinoma is based on clinical criteria, which can be controversial in some cases.

Assessing nodal status after NAC using US is highly subjective and often inaccurate [19]. In our study, physiological lymph nodes in the re-staging US examination (ycN0) were defined as those exhibiting no pathological signs, such as a longitudinal–transverse ratio of less than 1.5, loss of fatty hilus, or a cortical thickness greater than 3 mm. However, Pislair et al. demonstrated that strict evaluation using these criteria can result in overtreatment with ALND in up to 30% of patients. [20]. It is therefore recommended to interpret US results in the context of tumor phenotype, as its positive predictive value is sufficiently high only for luminal HER2-negative phenotypes, while it fails for TNBC and HER2-positive tumors [20]. This is evident in our results, as luminal HER2-negative tumors were not represented in the group of unnecessarily indicated dissections (ypN0), with one exception. It is also critical to consider whether the only persistent “suspected” or “pathological” node is the marked node that will be excised and examined, or whether multiple nodal pathologies are present. When multiple nodal pathologies are observed, greater caution is warranted, as TAD could theoretically carry a higher risk of false negativity for the marked node. In this regard, it may be problematic if markers that are not visible through imaging methods, such as carbon, are used for nodal marking. If a single persistent pathological node is observed during restaging US examination, it is not possible to determine whether it is the marked node or not.

A recently published study by the TAD Study Group highlights considerable variability in the criteria for indicating TAD worldwide, particularly regarding disease extent at initial presentation. Among surgeons using TAD, 27% perform this procedure irrespective of T-stage, while the majority limit its use to tumors up to T3. Regarding lymph node burden, most surgeons (42%) consider TAD appropriate for patients with up to three positive nodes, while 12% apply it regardless of the degree of axillary involvement [21]. From our cohort, five patients underwent ALND due to extensive initial involvement, with specific reasons being T4b stage or higher, or more extensive nodal involvement classified as N2 (matted nodes) or N3 (including supraclavicular node involvement). Of these five patients, four showed complete nodal regression prior to surgery based on US re-staging, while the fifth patient exhibited palpable tenderness in the axilla during clinical examination (without ultrasound examination). These findings demonstrate that even in locally advanced carcinomas, complete regression of axillary involvement can occur, and ALND may not always represent a rational approach here. This is particularly evident when originally affected nodes are located outside the dissected area (e.g., supraclavicular nodes), making “complete nodal clearance” through ALND unattainable. This perspective is supported by the study by Ng et al., which found no significant difference in the incidence of complete pathological axillary response between patients with low and high nodal burdens. Their analysis concluded that the biologic subtype correlated more strongly with axillary complete pathological response rates than nodal burden alone [22].

Apart from TAD with its inherent variabilities, SLNB should also be considered as an option for axillary lymph node staging after NAC. Two multicenter trials demonstrated that SLNB in patients initially cN+, who regressed to ycN0 after NAC, was associated with an unacceptably high FNR exceeding 10% [2,3]. However, long-term survival data related to this approach were not available in the literature at the time. One promising study recently published by Tinterri et al. concluded that SLNB could be a safe alternative to ALND in cN+ patients who regress to ycN0 after NAC, as it showed better survival rates in the SLNB group with similar axillary recurrence rates [23]. However, the limitations of this study include its retrospective design and the heterogeneity of the patient group. Therefore, future prospective studies are necessary to confirm these findings.

In two cases, the main reason for ALND was occult carcinoma, where the initial presentation involved carcinoma of mammary origin in the axillary nodes, but no abnormal findings were detected in the breast, including on magnetic resonance imaging. Given the very rare occurrence of this clinical entity, it does not seem realistic to organize a robust prospective study that would definitively answer the question of the best surgical approach. However, an extensive comparative study using data from the National Cancer Data Base found no difference in overall survival between patients who underwent ALND and those who had SLNB, provided they also received RT [24]. Nonetheless, we believe that, in principle, the surgical approach in the axilla may not differ from situations where the primary tumor in the breast is clearly diagnosed alongside nodal involvement. Therefore, it may be reasonable to consider TAD if signs of axillary regression are observed on ultrasound. However, it is important to note that in both of cases in our ypN0 cohort, although ultrasound restaging detected some degree of regression, it was not completely negative. In one case, a “suspect” node persisted (the marked node), and in the other, signs of regression were present, but two suspect nodes persisted, one of which was marked. Nevertheless, it is likely that if these had not been an occult carcinoma, TAD would have been indicated in both cases, or at least in the first case.

In six cases, it was not possible to determine the primary reason for indicating ALND from the available documentation. Of these, three cases involved patients initially diagnosed at a different institution, where different standards of pre-treatment diagnostics were applied (e.g., one case involved diagnosis through diagnostic surgical excision of a node, or the pathological nodes were not clipped at the time of diagnosis). In one case, the decision to perform ALND was influenced by the patient’s preference for a “more radical procedure” after being informed of the available options. In another case, the decision was based on preoperative lymphoscintigraphy, which did not show a peak of radioactivity in the axilla. As a result, the surgeon opted to proceed with ALND before even starting the surgery, despite the fact that the pathological node had been marked prior to treatment.

In the other six cases, ALND was performed because the patients participated in the IMpassion050 clinical trial (ClinicalTrials.gov NCT03726879). This study, which was exclusive to HER2-positive tumors, mandated ALND for all patients, regardless of the clinical effect of NAC. Of these six cases, re-staging US confirmed complete nodal regression in four patients, while nodal status remained uncertain in two cases. It can be assumed that, had these patients not been part of the study, at least those four individuals would have been indicated for TAD rather than ALND. This highlights the importance of considering the risk–benefit ratio in clinical trials, particularly in our specific case, where the risks of surgical axillary overtreatment may not have been justified by the potential benefits of the study drug therapy.

As mentioned in the introduction, adjuvant RT usually follows surgical treatment, regardless of its extent, in patients initially presenting with axillary metastases. In both cases from our ypN0 cohort where ALND was indicated due to contraindication to RT, the patients had a history of Hodgkin’s disease and had previously undergone RT. Given this history, a more radical procedure (ALND) was chosen for the axilla. In the case of prior RT to lymphatic regions, further irradiation options become limited. Overdosing could result in complications such as tissue necrosis, radiation ulcers, or fibrosis leading to lymphedema. Additionally, information about the previous irradiation conditions is often incomplete, and the fusion of irradiation plans to assess the risk of serious side effects from further irradiation is not feasible. In such cases, only small-volume palliative irradiation, with individual dosing depending on the prior RT dose, could be performed [7].

Finally, it must be acknowledged that in two cases, the sole reason for ALND appeared to be a misunderstanding in interpreting the report from the re-staging US examination. In these cases, although the radiologist described signs of regression in the lymph node area in the examination report, the parametrically stated conclusion in the categories of pathological/suspicious/negative selected the option “pathological nodes”. Subsequent clarification revealed that by categorizing the nodes as “pathological”, the radiologist was not indicating that signs of metastasis persisted, but rather referring to the fact that the nodes had been biopsied prior to treatment, with a finding of carcinoma metastasis. As such, the radiologist continued to categorize them as “pathological”.

Of course, our study has certain limitations, primarily because it is a retrospective review of a selected subgroup of patients from a prospectively maintained database. Ultrasonographic and histopathological diagnostics were not subject to central evaluation, and surgeries were performed by different surgeons. Additionally, the criterion for the minimum number of NAC cycles required was not consistently established, and some patients who prematurely discontinued NAC were included. However, this is not a major concern since the focus of the study is on patients in whom NAC was effective and who achieved nodal regression. Despite these limitations, the overall sample size (*n* = 560) is large, and all patients were treated at a single Comprehensive Cancer Center, ensuring consistency in procedures and diagnostic completeness. Furthermore, the indication for medical procedures, including surgery, was always discussed within a multidisciplinary team.

These results provide “real-world evidence”, which, in the absence of prospective randomized clinical trials specifically focused on breast cancer surgery, constitutes a significant part of our current knowledge. The goal of the study is not to provide definitively answers to the clinical questions raised, but rather to highlight specific patient groups who may still experience surgical axillary overtreatment, despite advances in modern systemic treatment and the era of TAD. The reduction in surgical radicality has been an ongoing process in breast cancer care over the past few decades, and we hope that new findings will soon emerge to guide its application—not only in typical patients meeting widely accepted entry criteria, but also in the less common subgroups we have sought to emphasize in our work.

## 5. Conclusions

This retrospective analysis, based on a prospectively maintained database at the Comprehensive Cancer Centre, identifies, through real-world evidence, subgroups of breast cancer patients treated with NAC who may suffer from surgical overtreatment in the axilla, even in the current modern era of TAD. These subgroups include patients with inflammatory carcinoma, locally advanced carcinoma, occult carcinoma, and those with persistent suspicious pathological nodes after NAC, as assessed by ultrasonography, particularly in HER2-positive and triple-negative breast cancer phenotypes. The study highlights potential directions for future research in breast cancer surgery.

## Figures and Tables

**Table 1:** Clinicopathological characteristics of patients with directly indicated ALND.

		Total (*n* = 118)	ypN0 (*n* = 44)	ypN+ (*n* = 74)	OR (95% CI)	*p* Value
Age Median (range)		55 (27, 85)	56 (27, 78)	54 (29, 85)	1.01 (0.99, 1.04)	0.344
cT	T1	13 (11%)	7 (54%)	6 (46%)	1.86 (0.43, 8.27)	0.405
	T2	48 (41%)	12 (25%)	36 (75%)	6.50 (2.10, 22.3)	0.002
	T3	17 (14%)	6 (35%)	11 (65%)	3.97 (1.03, 17.0)	0.051
	T4b	18 (15%)	4 (22%)	14 (78%)	7.58 (1.87, 37.0)	0.007
	T4d	19 (16%)	13 (68%)	6 (32%)	Ref	
	TX	3 (2.5%)	2 (67%)	1 (33%) ^⌂^	NS	
cN	* N0/1	1 (0.8%)	0 (0%)	1 (100%)	NS	
	N1	93 (79%)	38 (41%)	55 (59%)	Ref	
	N2	14 (12%)	1 (7.1%)	13 (93%)	8.98 (1.68, 167)	0.038
	N3	10 (8.5%)	5 (50%)	5 (50%)	0.69 (0.18, 2.64)	0.579
Phenotype	Luminal A	4 (3.4%)	1 (25%) □	3 (75%)	9.60 (0.99, 221)	0.073
	Luminal B HER2 negative	33 (28%)	1 (3.0%) ^#^	32 (97%)	102 (15.9, 2073)	<0.001
	Luminal B HER2 positive	18 (15%)	8 (44%)	10 (56%)	4.00 (1.06, 16.9)	0.047
	** Luminal B HER2 positive/negative	1 (0.8%)	0 (0%)	1 (100%)	NS	
	Non-luminal HER2 positive	21 (18%)	16 (76%)	5 (24%)	Ref	
	Triple-negative breast cancer	41 (35%)	18 (44%)	23 (56%)	4.09 (1.32, 14.5)	0.019
Type of breast surgery	Breast-conserving surgery	30 (25%)	7 (23%)	23 (77%)	Ref	
	Mastectomy	87 (74%)	36 (41%)	51 (59%)	0.43 (0.16, 1.07)	0.082
	No surgery	1 (0.8%)	1 (100%)	0 (0%)		
ypT	ypT0	29 (25%)	22 (76%)	7 (24%)	Ref	
	ypTis	12 (10%)	8 (67%)	4 (33%)		
	ypT1	38 (32%)	10 (26%)	28 (74%)	7.64 (2.91, 21.7)	<0.001
	ypT2	27 (23%)	0 (0%)	27 (100%)	46.4 (11.6, 317)	<0.001
	ypT3	4 (3.4%)	1 (25%)	3 (75%)		
	ypT4b	5 (4.2%)	1 (20%)	4 (80%)		
	X	3 (2.5%)	2 (67%)	1 (33%)	NS	
Number of examined lymph nodes Median (range)		12 (1, 37)	11 (1, 37)	12 (5, 30)	1.07 (1.0, 1.17)	0.086

* At the time of treatment initiation cN0 progression to cN1 occurred during NAC. ** Two tumors of different phenotypes, HER2+ and HER2−, were present in the same breast. ^⌂^ Considered synchronous bilateral cancer at the time of surgery: tumor in the left breast and pathological nodes in both axillae. On the right—initially categorized as occult carcinoma; however, after a definitive examination of surgical resections (bilateral modified radical mastectomy), histopathologically confirmed as extensive metastatic carcinoma of the left breast spreading to both axillae, thus representing distant metastasis in the right axilla; this was also consistent with subsequent clinical course—rapid progression in various locations and distant organs. □ Synchronous bilateral cancer—Luminal A with a HER2-positive T3N0 tumor contralaterally; NAC was indicated due to this HER2-positive tumor, yet complete histopathological regression occurred even in the case of lobular carcinoma with a Luminal A phenotype. HER2-positive carcinoma is not included in the target population because it was indicated to SLNB. ^#^ The patient was classified as ypN0, but it was not a complete regression, because ITC and multifocal lymphovascular invasion were found in three nodes. NS—not specified; Ref—reference category.

**Table 2:** The reasons for ALND in ypN0 patients.

Reason for ALND	Number of Cases
Inflammatory carcinoma	13 (29.5%)
Persistent pathological nodes on re-staging US examination	8 (18.2%)
Participation in a drug clinical trial	6 (13.6%)
Combination of multiple “minor” factors	6 (13.6%)
Locally advanced breast cancer (LABC) at initial presentation (T4bN2, T4bN1, T3N2, T3N3, T2N3)	5 (11.4%)
Occult carcinoma (supported by re-staging ultrasound examination)	2 (4.5%)
Contraindications to adjuvant radiotherapy	2 (4.5%)
Misinterpretation of ultrasound examination	2 (4.5%)
Total	44 (100%)

## Data Availability

All data generated or analyzed during this study are included in this article. Further enquiries can be directed to the corresponding author.

## Abbreviations

The following abbreviations are used in this manuscript:

NAC	Neoadjuvant Chemotherapy
TAD	Targeted Axillary Dissection
ALND	Axillary Lymph Node Dissection
SLNB	Sentinel Lymph Node Biopsy
FNR	False-Negative Rate
US	Ultrasound
RT	Radiotherapy
HER2	Human Epidermal Growth Factor Receptor 2
TNBC	Triple-Negative Breast Cancer

The TNM classification used in the manuscript follows the 8th edition of the *TNM Classification of Malignant Tumours* by James D. Brierley et al. [15].
